# A mechanics-based perspective on the pressure-cross-sectional area loop within the esophageal body

**DOI:** 10.3389/fphys.2022.1066351

**Published:** 2023-01-09

**Authors:** Guy Elisha, Sourav Halder, Dustin A. Carlson, Peter J. Kahrilas, John E. Pandolfino, Neelesh A. Patankar

**Affiliations:** ^1^ Department of Mechanical Engineering, McCormick School of Engineering, Northwestern University, Evanston, IL, United States; ^2^ Theoretical and Applied Mechanics Program, McCormick School of Engineering, Northwestern University, Evanston, IL, United States; ^3^ Division of Gastroenterology and Hepatology, Feinberg School of Medicine, Northwestern University, Chicago, IL, United States

**Keywords:** sphincter, esophagus, peristalsis, pressure-area hysteresis loop, functional luminal imaging probe, mechanical states

## Abstract

**Introduction:** Plotting the pressure-cross-sectional area (P-CSA) hysteresis loops within the esophagus during a contraction cycle can provide mechanistic insights into esophageal motor function. Pressure and cross-sectional area during secondary peristalsis can be obtained from the functional lumen imaging probe (FLIP). The pressure-cross-sectional area plots at a location within the esophageal body (but away from the sphincter) reveal a horizontal loop shape. The horizontal loop shape has phases that appear similar to those in cardiovascular analyses, whichinclude isometric and isotonic contractions followed by isometric and isotonic relaxations. The aim of this study is to explain the various phases of the pressurecross-sectional area hysteresis loops within the esophageal body.

**Materials and Methods:** We simulate flow inside a FLIP device placed inside the esophagus lumen. We focus on three scenarios: long functional lumen imaging probe bag placed insidethe esophagus but not passing through the lower esophageal sphincter, long functional lumen imaging probe bag that crosses the lower esophageal sphincter, and a short functional lumen imaging probe bag placed in the esophagus body that does not pass through the lower esophageal sphincter.

**Results and Discussion:** Horizontal P-CSA area loop pattern is robust and is reproduced in all three cases with only small differences. The results indicate that the horizontal loop pattern is primarily a product of mechanical conditions rather than any inherently different function of the muscle itself. Thus, the distinct phases of the loop can be explained solely based on mechanics.

## 1 Introduction

A healthy functioning esophagus transports swallowed material from the mouth towards the stomach through peristaltic contraction (Regan et al., 2012; Jain et al., 2019). Patients with systemic sclerosis, achalasia, dysphagia, and other motor disorders have impaired esophageal peristalsis (Kahrilas, 2008; Shaheen et al., 2010; Gregersen et al., 2011; Aziz et al., 2016). Thus, understanding esophageal motor function and investigating the contractile behavior of esophageal smooth muscles can help in identifying esophageal disease progression and develop diagnostic tools. Extensive computational studies have been performed in recent years on the mechanics of esophageal transport (Kou et al., 2017a; Kou et al., 2017b; Kou et al., 2018; Kou et al., 2017c; Acharya et al., 2022; Halder et al., 2021; Elisha et al., 2022a; Acharya et al., 2021c; Acharya et al., 2021a; Elisha et al., 2021; Liao et al., 2020) and emptying as well as the state of various esophageal diseases (Acharya et al., 2021b; Halder et al., 2022a; Halder et al., 2022c; Halder et al., 2022b).

In recent years, several studies used pressure-cross-sectional area (P-CSA) relationship during esophageal contraction cycle to investigate esophageal wall function (Villadsen et al., 2001; Gregersen et al., 2011; Liao et al., 2013; Leibbrandt et al., 2016; Elisha et al., 2021). This approach is borrowed from cardiovascular analyses, which uses pressure-volume (P-V) plots to study dynamic motor activity and explain the pumping mechanism of the left ventricle and its dysfunction (Gregersen et al., 2007; Gregersen et al., 2011; Liao et al., 2013; Gregersen and Lo, 2018).

**FIGURE 1:**
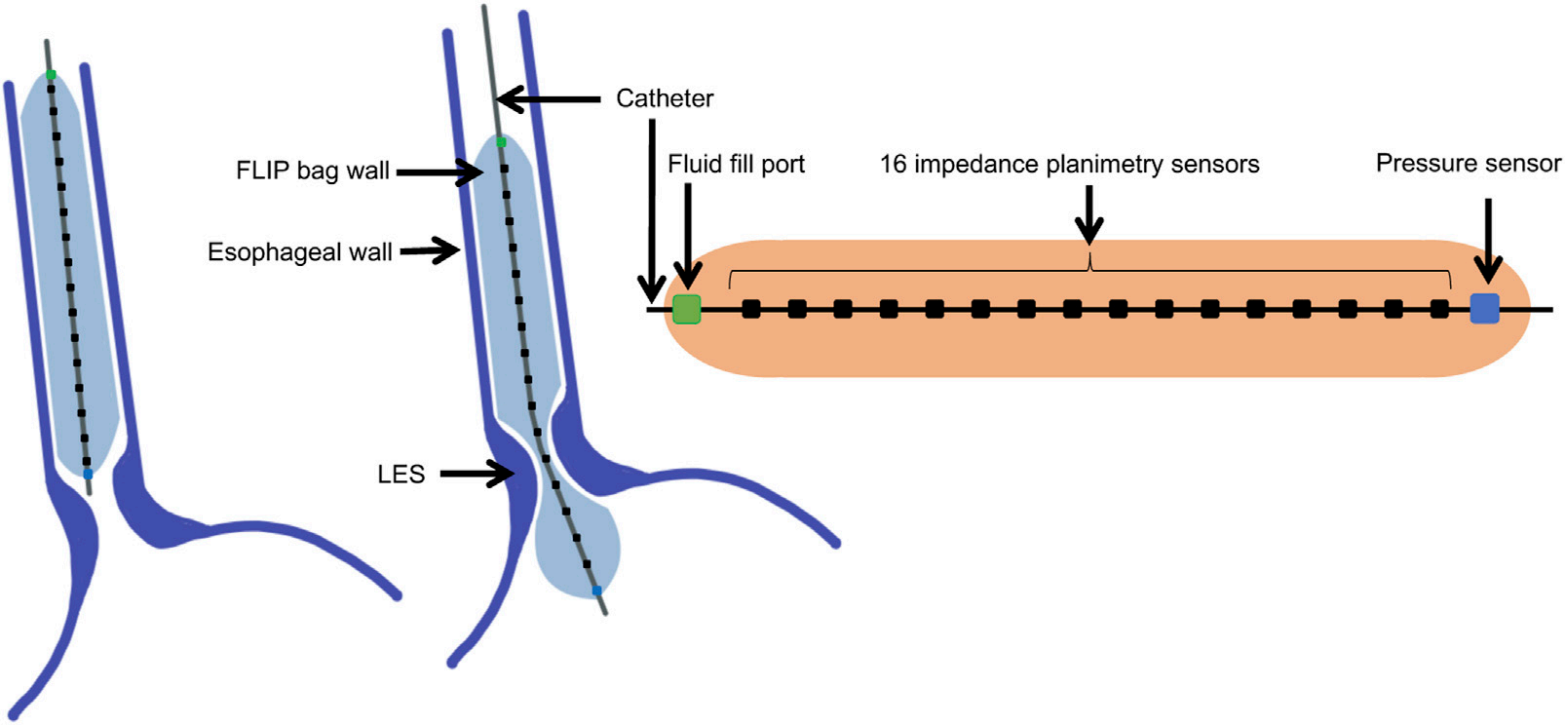
Diagram showing the FLIP bag and catheter assembly placed within the esophagus, passing through the LES or not. The bag used in this study is 16 cm long and 22 mm diameter. The catheter measures CSA at 16 location and one distal pressure measurement at each time instant.

Tracking the pressure and CSA during a distention-induced contraction cycle of the esophagus was made possible by the functional lumen imaging probe (FLIP) (Liao et al., 2014; Moonen and Boeckxstaens, 2014; Lottrup et al., 2015). The FLIP device, illustrated in Figure 1, consists of a catheter surrounded by a fluid filled bag, placed inside the esophagus. The filled bag results in mechanical distention, which induces secondary peristalsis in the esophagus (Clarke et al., 2020). The response of the esophagus wall to the distention is captured by the FLIP in the form of CSA readings at 16 locations and one distal pressure measurement as a function of time (Carlson et al., 2015; Carlson et al., 2016). The FLIP in the esophagus can be placed such that it passes through the lower esophageal sphincter (LES), as in the middle illustration in Figure 1, or not, as in the left illustration in Figure 1.

**FIGURE 2:**
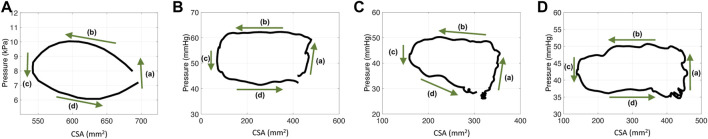
P-CSA relationships in the esophagus body for four different contraction cycles with different experimental setups. **(A)** P-CSA extracted from a 4-cm long FLIP obtained by Gregersen et al. (2011). **(B)** and **(C)** P-CSA extracted from a 16-cm long FLIP at 60 ml, where the FLIP bag passes through the LES. These hystereses are plotted from data recorded at a sensor located away from the LES. **(D)** P-CSA extracted from a 16-cm long FLIP at 60 ml, where the FLIP bag does not cross the LES.

As reported by Gregersen et al. (2011), the P-CSA hysteresis loop, at any location within the esophagus body (i.e., away from the lower esophageal sphincter or the LES), during distention-induced secondary peristalsis reveals a horizontal loop shape, presented in Figure 2A. This loop pattern looks similar to the one obtained from the left ventricle, and therefore, the loop was explained analogously. The esophageal P-CSA hysteresis loop was divided into four phases, each named analogously after the corresponding phase on the P-V loop plotted for the left ventricle. These phases are isometric contraction, isotonic contraction, isometric relaxation, and isotonic relaxation (Gregersen et al., 2011). Similar terminology was used by others Leibbrandt et al. (2016); Omari et al. (2015) when analyzing P-CSA hysteresis loop within the esophagus body or at the upper esophageal sphincter using a different measuring device.

While the resulting P-CSA loop within the esophagus body during the FLIP experiment and the P-V loop in the left ventricle have a similar pattern, the mechanical functioning is different. For example, the volume inside the ventricle changes through the cardiac cycle whereas the volume inside the esophagus during secondary peristalsis in a FLIP device is constant. Here, we aim to provide a mechanics-based explanation for the P-CSA loop within the esophagus body. Specifically, we show that the various phases in the loop shape are a result of the mechanical conditions (muscle contraction, boundary condition, and geometric configuration) rather than any inherently different function of the muscle itself.

### 1.1 Pressure-CSA loops within the esophagus body

Figure 2 presents four plots of the pressure within the esophageal body as a function of wall CSA during contraction cycles from four different experiments. Figure 2A displays the results obtained by Gregersen et al. (2011). In their work, the FLIP bag was 4 cm long and the maximum bag diameter was 5 cm. The middle of the bag was placed 7 cm above the LES and did not pass through it. Figures 2B, C display two examples of P-CSA loops obtained by a 16 cm FLIP bag at 60 ml bag fill, where the bag passes through the LES. Lastly, the loop plotted in Figure 2D is extracted by a 16 cm FLIP bag at 60 ml bag fill, where the bag does not cross the LES.

The data for Figures 2B–D was collected at the Esophageal Center of Northwestern between November 2012 and October 2018, using EndoFLIP^®^ (EF-322N; Medtronic, Inc., Shoreview, MN, United States) (Acharya et al., 2021a; Carlson et al., 2021). We examined a total of 24 randomly selected, asymptomatic volunteers for the cases where the FLIP passes through the LES, and two cases where it does not. Informed consent was obtained for subject participation; they were paid for their participation. The study protocol was approved by the Northwestern University Institutional Review Board. Additional details on the data collection process and subject cohort selection is available in (Lin et al., 2013; Carlson et al., 2016; Carlson et al., 2021; Acharya et al., 2021a). Since FLIP provides only one distal pressure reading, pressure at different locations along the esophagus length was calculated using the distal pressure, CSA measurements, the mass conservation equation, and the momentum equation (discussed in Section 2.1), as proposed by Halder et al. (2021).

As Figure 2 reveals, the P-CSA hysteresis plotted at a location within the esophagus body displays a loop of average horizontal orientation. This is opposed to the loops of positive or negative slope orientation which have been reported at sphincter locations of different organs (Zifan et al., 2019; Elisha et al., 2022b). A loop in considered horizontal when the magnitude of the slope of its best fit line for the point cloud representing the hysteresis is such that |slope| < .1. The point cloud is obtained by discretizing the loop into equal CSA segments (*∆A*) and finding the corresponding pressure values. In the positive and negative slope loops at sphincter locations of different organs, the slopes are clearly greater in magnitude than the .1 threshold used above to define a horizontal loop.

On each plot in Figure 2, the four phases identified by Gregersen et al. (2011) are marked. Gregersen et al. (2011) refer to phases 1 and 2 as contraction, while phases 3 and 4 as relaxation. To explain these phases, we reproduce esophageal secondary peristalsis using simulations, discussed next.

## 2 Materials and methods

In our previous studies, we developed a one dimensional (1D) model of a flow inside an elastic tube closed on both ends to imitate a flow inside a FLIP device (Elisha et al., 2022a; Elisha et al., 2021; Acharya et al., 2021c; Halder et al., 2022c). Our goal was to study the relation between tube properties, fluid properties, muscle activation pattern, and their effect on pressure and CSA of the tube. In this section, we summarize the mathematical model.

### 2.1 Governing equations in 1D

The 1D mass and momentum conservation equations are

$$\frac{\partial A}{\partial t}+\frac{\partial \left(Au\right)}{\partial x}=0,$$ (1)

and

$$\frac{\partial u}{\partial t}+u\frac{\partial u}{\partial x}=-\frac{1}{\rho }\frac{\partial P}{\partial x}-\frac{8\pi \mu u}{\rho A},$$ (2)

respectively, which were derived by Ottesen (2003). Here, *A*(*x*, *t*), *u*(*x*, *t*), *P*(*x*, *t*), *ρ* and *μ* are the tube CSA, fluid velocity (averaged at each cross-sectional area), pressure inside the tube, fluid density, and fluid viscosity, respectively. To complete the system, we relate pressure and CSA by a linear constitutive equation of the form

$$P=K_{e}\left(\frac{A\left(x,t\right)}{A_{o}\theta \left(x,t\right)}-1\right)+P_{o}.$$ (3)

This relation, also known as the “Tube Law,” was derived by Whittaker et al. (2010) and validated experimentally by Kwiatek et al. (2011). In the equation above, *P*_*o*_ is the outside pressure, *K*_*e*_ is tube stiffness, and *A*_*o*_ is the undeformed reference area (CSA of the tube when *P* = *P*_*o*_) (Elisha et al., 2022a; Elisha et al., 2021; Elisha et al., 2022b; Acharya et al., 2021c). Lastly, to mimic muscle peristaltic contraction, we vary the reference area sinusoidally by multiplying *A*_*o*_ by an activation function *θ*(*x*, *t*) (Ottesen, 2003; Manopoulos et al., 2006; Bringley et al., 2008; Acharya et al., 2021c; Elisha et al., 2022a). As Eq. 3 suggests, contraction implies reduction of the tube’s reference CSA (*θ* < 1), and relaxation is associated with increase in the reference CSA (*θ* > 1). When *θ* = 1, neither contraction nor relaxation are present. Note that in the simulations described in this report, *θ* is either less than or equal to 1. The expression for *θ*(*x*, *t*) is discussed in Section 2.3.

### 2.2 Non-dimensional dynamic equations

We non-dimensionalize the dynamic system using

$$A=\alpha A_{o},\;t=\tau \frac{L}{w_{c}},\;u=Uw_{c},P=pK_{e},\;\text{and}\;x=\chi L,$$ (4)

to reduce the number of independent variables. The terms *α*, *τ*, *U*, *p*, and *χ* are the non-dimensional variables of area, time, velocity, pressure, and position, respectively (Acharya et al., 2021c; Elisha et al., 2022a). The dimensional wave speed and tube length are $w_{c}$ and *L*, respectively.

Using the above, the mass conservation, momentum conservation, and tube law equations can be written as

$$\frac{\partial \alpha }{\partial \tau }+\frac{\partial \left(\alpha U\right)}{\partial \chi }=\epsilon {\left(\frac{\alpha }{\theta }\right)}_{\chi \chi },$$ (5)

$$\frac{\partial U}{\partial \tau }+U\frac{\partial U}{\partial \chi }+\psi \frac{\partial p}{\partial \chi }+\beta \frac{U}{\alpha }=0,\;\text{and}$$ (6)

$$p=\left(\frac{\alpha }{\theta }-1\right)-\eta \frac{\partial \left(\alpha U\right)}{\partial \chi },$$ (7)

respectively. The terms $\psi =K_{e}/\left(\rho {w_{c}}^{2}\right)$ and *β* = 8*πμL*/(*ρA*_*o*_ $w_{c}$ ) are non-dimensional stiffness and viscosity parameters, respectively. Notice the we introduce a smoothing term $\left(\epsilon {\left(\alpha /\theta \right)}_{\chi \chi }\right)$ to the right-hand side of Eq. 5 which improves convergence speed and reduces computational time (Acharya et al., 2021c; Elisha et al., 2022a). Additionally, we introduced a damping term $\left(\eta \frac{\partial \left(\alpha U\right)}{\partial \chi }\right)$ to the pressure Eq. 7 which helps in regularizing the system, thus stabilizing the numerical solution. The non-dimensional damping parameter *η* is defined by *η* = (*Y* $w_{c}$ *A*_*o*_)/(*K*_*e*_ *L*), where *Y* is the damping coefficient (Acharya et al., 2021c; Elisha et al., 2022a).

### 2.3 Peristaltic wave input and active relaxation

**FIGURE 3:**
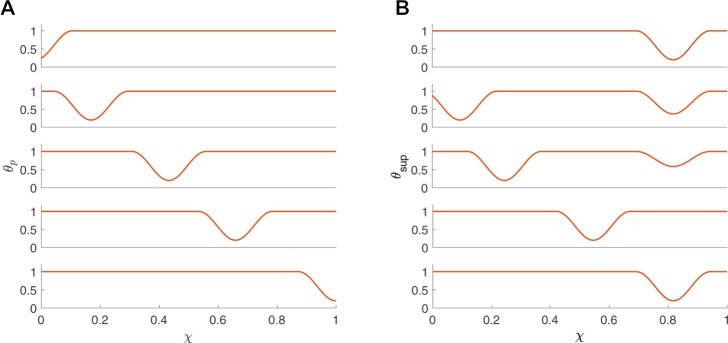
Activation functions **(A)** *θ*_*p*_ and **(B)** *θ*_sup_ **(B)** plotted as a function of *χ* at five consecutive time instants.

The activation function (*θ*) can take different forms, and implementing it as described in Section 2.1 is a common approach when the external activation pressure at a specific location varies sinusoidally with time. As discussed in Elisha et al. (2022a), the *θ* pattern chosen for the model in this study aims to mimic clinical observations of esophageal secondary peristalsis reported by Goyal and Chaudhury (2008) and Crist et al. (1984). When the FLIP bag does not pass through the sphincter, we define the activation function as simply a traveling sinusoidal wave with constant amplitude, wavelength and wave speed of the form

$$\theta_{p}\left(\chi ,\tau \right)=\left\{\begin{array}{cc} 1-\left(\frac{1-\theta_{c}}{2}\right)\left[1\;+\sin\left(\frac{2\pi }{\text{w}}\left(\chi -\tau \right)+\frac{3\pi }{2}\right)\right],\; & \tau -\text{w}\leq \chi \leq \tau \\ 1,\; & \text{otherwise,} \end{array}\right.$$ (8)

where w is the non-dimensional width of the peristaltic wave (dimensional width/*L*) and *θ*_*c*_ is the maximum contraction strength (wave amplitude = *θ*_*c*_) (Acharya et al., 2021c; Elisha et al., 2022a). Figure 3A presents *θ*_*p*_(*χ*, *τ*) at five consecutive time instants. Given that an average esophageal peristaltic wave is about 3–4 cm wide, w = .25 for a 16 cm bag and w = .75 for a 4 cm bag. In the activation function presented in Figure 3A, w = .25.

For the scenario where the FLIP bag passes through the LES, the activation function is defined as a superposition of *θ*_*p*_(*χ*, *τ*) with *θ*_LES_(*χ*, *τ*), where *θ*_LES_(*χ*, *τ*) is responsible for the initial tone and relaxation of the LES, such that

$$\theta_{\text{sup}}\left(\chi ,\tau \right)=\theta_{p}\left(\chi ,\tau \right)+\theta_{\text{LES}}\left(\chi ,\tau \right).$$ (9)

The function *θ*_LES_(*χ*, *τ*) is defined by

$$\theta_{\text{LES}}\left(\chi ,\tau \right)=\left\{\begin{array}{cc} \frac{\theta_{r}\theta_{R}\left(\tau \right)-1}{2}\left[1\;+\sin\left(\frac{2\pi }{{\text{w}}_{\text{LES}}}\left(\chi -\chi_{2}\right)+\frac{3\pi }{2}\right)\right],\; & \chi_{1}\leq \chi \leq \chi_{2}\\ 0,\; & \text{otherwise,} \end{array}\right.$$ (10)

where *θ*_*r*_ is the LES tone, w_LES_ is the non-dimensional width of the LES, *χ*_1_ and *χ*_2_ are the boundaries of the LES segment (*χ*_2_ > *χ*_1_ and w_LES_ = *χ*_2_−*χ*_1_), and

$$\theta_{R}\left(\tau \right)=\left\{\begin{array}{cc} 1,\; & \tau \leq \tau_{i}\\ 1+\frac{1+\frac{1}{\theta_{r}}}{\tau_{m}-\tau_{i}}\left(\tau -\tau_{i}\right),\; & \tau_{i}<\tau \leq \tau_{m}\\ \frac{1}{\theta_{r}},\; & \tau_{m}<\tau . \end{array}\right.$$ (11)

In the equation above, *τ*_*i*_ and *τ*_*m*_ are time instants, where *τ*_*i*_ marks the beginning of LES relaxation and *τ*_*m*_ marks the end of the relaxation process (Elisha et al., 2021).

### 2.4 Initial and boundary conditions

At *τ* = 0, the fluid inside the tube is at rest, thus, its velocity (*U*) is equal to zero for all *χ* (Acharya et al., 2021c; Elisha et al., 2022a). Additionally, it implies that the pressure in the tube is uniform (typically positive), which according to the tube law indicates that *α*/*θ* (at *τ* = 0) is a constant (= *S*_IC_). Consequently, the initial condition can be written as

$$U\left(\chi ,\tau =0\right)=0\;\;\alpha \left(\chi ,\tau =0\right)=S_{\text{IC}}\theta \left(\chi ,\tau =0\right).$$ (12)

The velocity boundary condition is simply

$$U\left(\chi =0,\tau \right)=0\;\text{and}\;U\left(\chi =1,\tau \right)=0,$$ (13)

since the tube is closed at both ends. The boundary condition for *α* is not as straightforward, but can be derived by plugging Eq. 7 and the velocity condition in Eq. 13 into Eq. 6, which yields

$${\left.\frac{\partial }{\partial \chi }\left(\frac{\alpha }{\theta }\right)\right|}_{\chi =0,\tau }=0\;and\;{\left.\frac{\partial }{\partial \chi }\left(\frac{\alpha }{\theta }\right)\right|}_{\chi =1,\tau }=0.$$ (14)

This condition is constructed by assuming that *η* ≈ 0. However, as explained by Acharya et al. (2021c), the effects due to this damping at the boundary are negligible for small *η*.

### 2.5 Numerical implementation

The system is solved using the MATLAB pdepe function. The numerical solution and the 1D simplification of peristaltic flow presented in this report was validated in Acharya et al. (2021c), where they compared the 1D simulation results with an equivalent 3D immersed boundary simulation (Peskin, 2002).

In previous studies, a large range of possible values for the different parameters were examined to conduct a parametric study. For example, although typical clinical values correspond to small *β* (order 1) and large *ψ* (order 10^3^), analyzing simulations only within this range means only capturing scenarios in which viscous effects are negligible. Therefore, larger *β* and smaller *ψ* were also considered (Elisha et al., 2022a; Elisha et al., 2021; Acharya et al., 2021c). However, based of the findings summarized in Elisha et al. (2022a) and Elisha et al. (2021), we choose to focus on simulations with parameter values listed in Table 1.

**TABLE 1:** List of non-dimensional parameters.

Symbol	Values	Definition
β	100	Dimensionless strength of viscous effects (inverse of Reynolds number)
*ψ*	100, 2400	Dimensionless rigidity of the elastic tube (inverse of Cauchy number)
(*τ*_*m*_−*τ*_*i*_)	0.6	Dimensionless LES relaxation time
*θ*_*c*_	0.2	Peristaltic contraction strength
*θ*_*r*_	0.2	LES contraction strength
w	0.25, 0.75	Width of peristaltic wave
w_LES_	0.25	LES width
*S*_IC_	2.0	Constant area that depends on the volume of the bag (*α*(*τ* = 0)/*θ*(*τ* = 0))

## 3 Results and discussion

Clinical data reveal that the P-CSA hysteresis loop during a single contraction cycle at a location within the esophageal body recorded by a FLIP device results a horizontal loop shape (Figure 2). To explain the underlying mechanisms of this contraction pattern by analyzing the different phases of these loops, we simulated different contraction cycles, as described in the previous section. In this section, we display and discuss the simulation results by plotting the P-CSA hysteresis for different scenarios. We focus on the horizontal loop orientation obtained at the esophageal body using a 16 cm FLIP.

**FIGURE 4:**
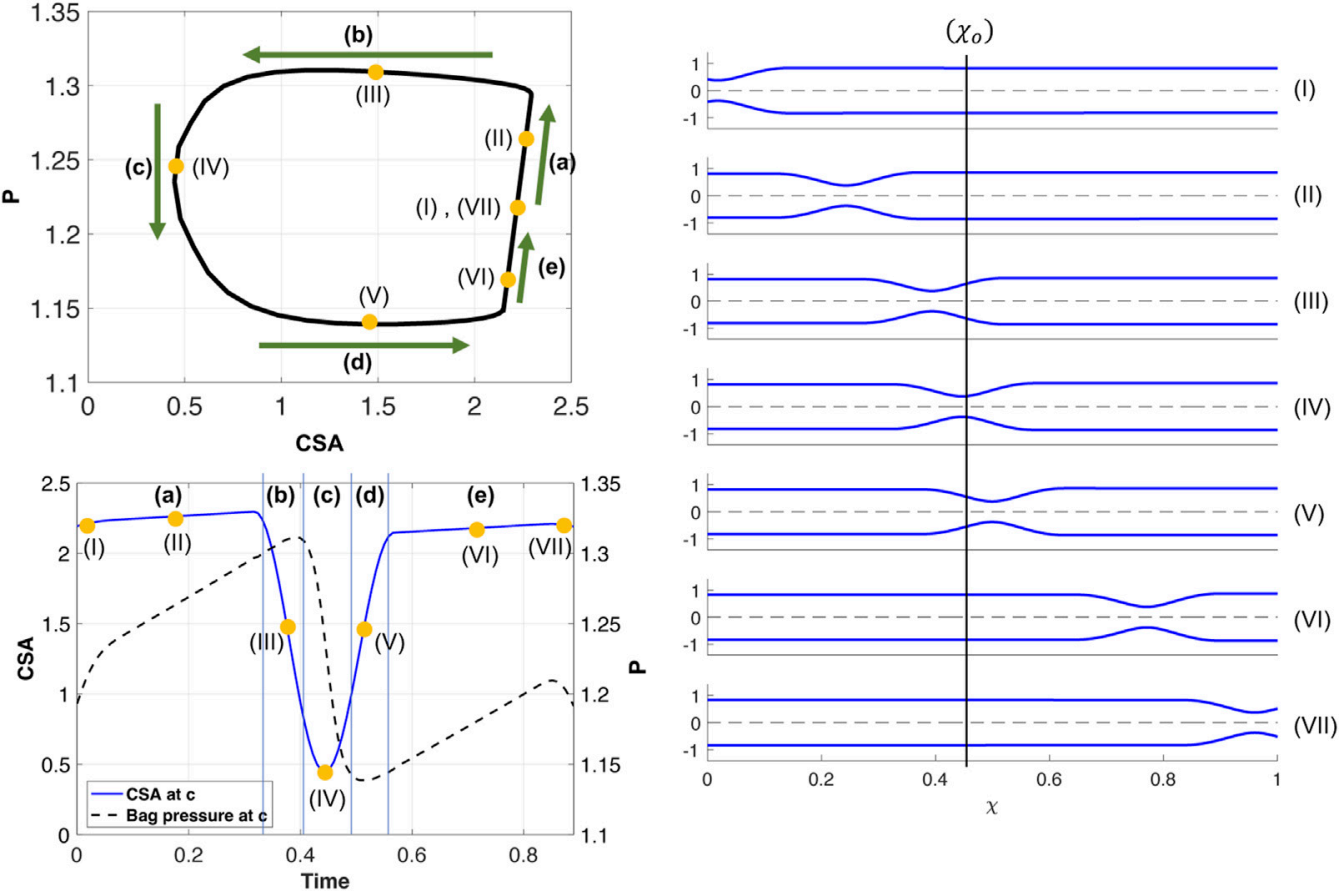
Simulation results for a single contraction cycle with muscle activity defined by a traveling peristaltic wave (*θ*_*p*_). Top left: P-CSA hysteresis loop at *χ*_*o*_. Bottom left: P and CSA vs. time at *χ* = *χ*_*o*_. Right: tube shape at seven consecutive instants, ordered chronologically.

Figure 4 presents the results of a simulation of an esophageal contraction cycle using a 16 cm long FLIP which does not pass through the LES. The activation function is *θ*_*p*_ with the input parameters listed in Table 1, w = .25, and *ψ* = 2400. The resulting tube shape at seven consecutive time instants are presented on the right and the location where the esophagus body loop is plotted is marked *χ* = *χ*_*o*_. The P-CSA loop at that location is plotted on the top left, and the pressure and CSA as a function of time are plotted on the bottom left. As the figure shows, a horizontal loop emerges.

**FIGURE 5:**
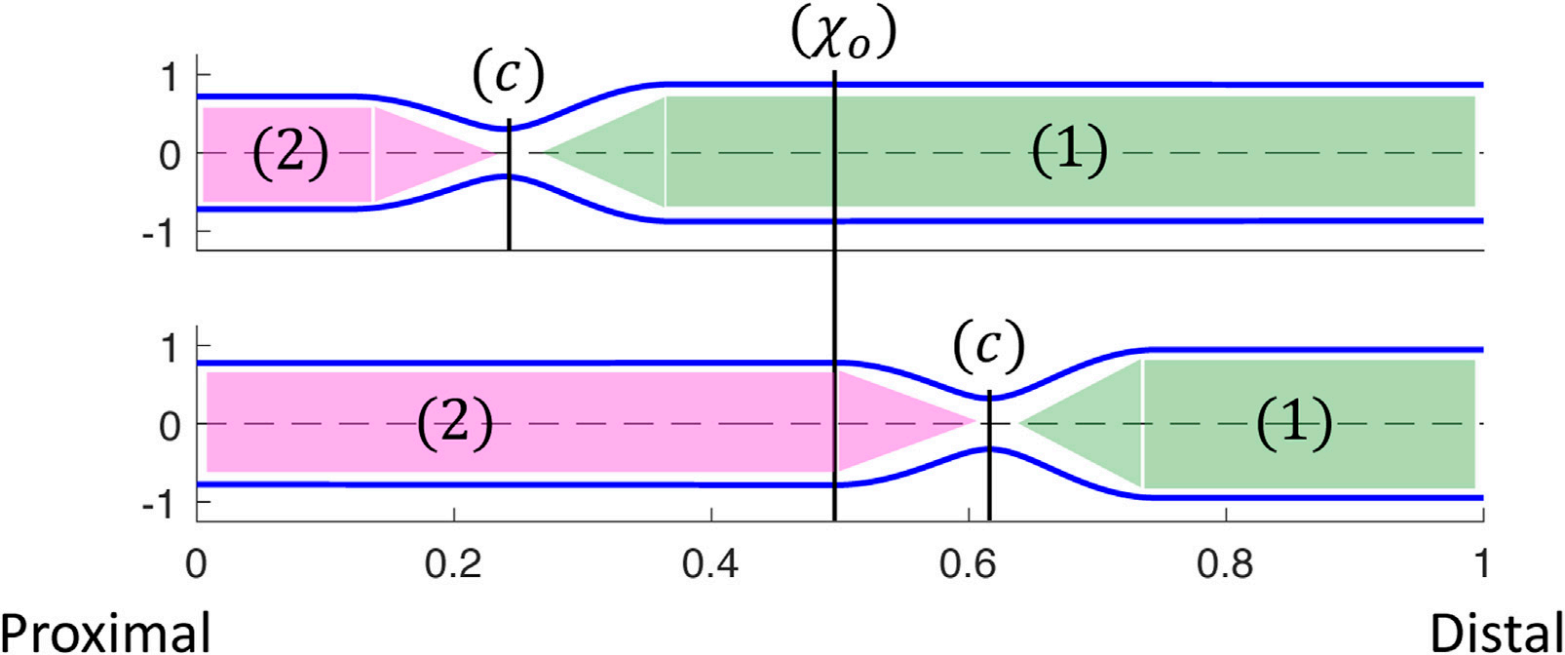
Two instants showing the corresponding tube shapes from the same simulation. The locations of point *χ* = *c*, regions 1 and 2, and how they change over time are depicted. P-CSA is plotted at *χ* = *χ*_*o*_ and its location does not change with time.

As in the prior clinical analysis by Gregersen et al. (2011), we divide the loop into phases **(a–e)** (Figure 4), which will help us understand the underlying mechanics causing the horizontal loop shape. To explain the different phases, we use the diagram in Figure 5. The diagram presents the shape of the elastic tube at two time instants during a contraction cycle. In the simulation, the wave travels from left to right (proximal to distal). The P-CSA loop is plotted (Figure 4) at the point *χ* = *χ*_*o*_, which is static. Point *χ* = *c* is where the maximum contraction of the advancing wave takes place (*θ* = *θ*_*c*_), and it moves with time. Regions 1 and 2 refer to the regions distal and proximal to the contraction, respectively.

As the peristalsis wave travels down the deformable tube, it pushes the fluid forward, such that the CSA, *A*_1_, at region 1 is greater than the CSA, *A*_2_, at region 2 (Acharya et al., 2021c; Elisha et al., 2022a). Therefore, the pressure, *p*_1_, at region 1 is also greater than the pressure, *p*_2_, at region 2. The minimum CSA, *A*_*c*_, is at *χ* = *c*. Thus, when *χ*_*o*_ is distal to the contraction, $p_{\chi_{o}}\approx p_{1}$ , and when *χ*_*o*_ is proximal to the contraction, $p_{\chi_{o}}\approx p_{2}$ , where $p_{\chi_{o}}$ is the pressure at *χ* = *χ*_*o*_.

The tube shape and the relation between *A*_1_, *A*_2_, and *A*_*c*_ were described by in our prior work (Acharya et al., 2021c; Elisha et al., 2022a). As the advancing contraction wave travels down the deformable tube, a competition between the elastic forces generated due to deformation of the tube wall and resistance to flow through the narrowest part of the contraction is created. When tube stiffness is high and fluid viscosity is relatively low, the tube wall resists deformation and forces the fluid that was displaced by the peristaltic wave to flow back through the contraction. In this scenario, the energy that is required to expand the tube walls is greater than the energy required to overcome viscous resistance across the contraction. Yet, *A*_1_ > *A*_2_, even if the difference between the two is small. When tube stiffness is relatively low and fluid viscosity is relatively high, an opposite phenomenon occurs. In this scenario, the resistance to flow is high, and therefore it is favorable for the tube wall at region 1 to expand and accommodate for the displaced fluid. In this second scenario, *A*_1_ is much greater than *A*_2_. With the above understanding, the different phases, as marked in Figure 4 are discussed next.

**FIGURE 6:**
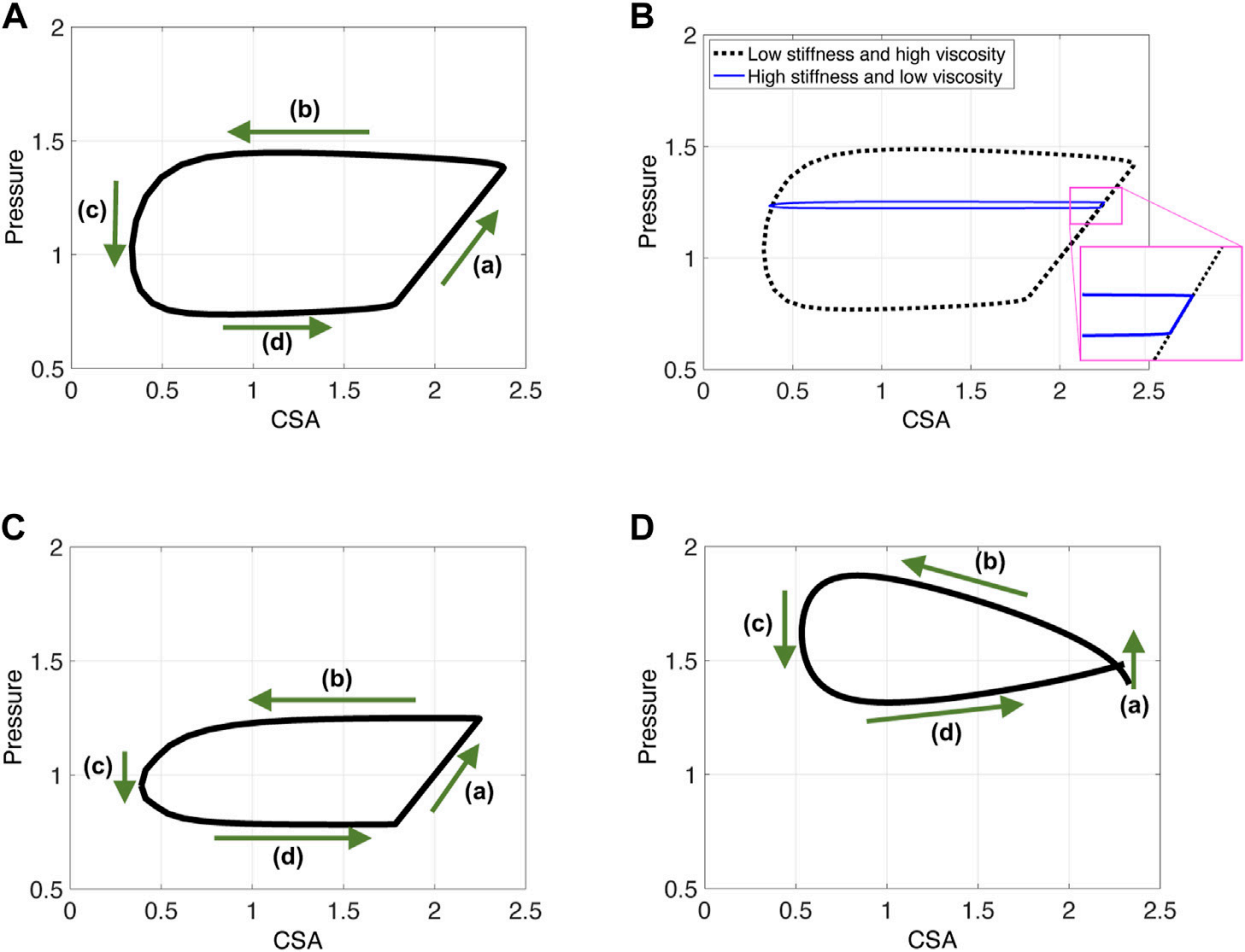
P-CSA plots obtained from three different contraction cycle simulations. Input parameters are as listed in Table 1 and *ψ* = 100. **(A, B)** The FLIP bag does not pass through the LES, w = .25, **(C)** the FLIP bag passes through the LES, w = .25, and **(D)** simulation results for a short FLIP bag, w = .75.

(a) As the contraction cycle starts, the peristaltic wave pushes the fluid forward, increasing the pressure distal to the contraction, which is also where point *χ*_*o*_ is located (Elisha et al., 2022a). Because fluid is displaced, CSA also increases at *χ* = *χ*_*o*_, alongside pressure. Due to the high tube stiffness (*ψ* = 2400), the increase in pressure and CSA is small. When viscosity increases and/or tube stiffness decreases, it is favorable for the tube to expand and accommodate for the displaced fluid. Thus, the line in phase **(a)** is longer as the change in pressure and CSA is more prominent, as seen in Figure 6A. Figure 6B displays a visual representation of the affect wall stiffness and fluid viscosity have on the loop, and specifically stage **(a)**. Phase **(a)** in esophageal FLIP experiment is not perfectly vertical or isometric (Figures 2, 4).
(b) When the contraction wave approaches *χ*_*o*_, the CSA at this location starts to decrease due to the contraction (i.e., *θ*(*χ*_*o*_, *τ*) decreases). However, during phase **(b)** point *χ*_*o*_ is still distal to the minimum area at *c*, and *θ*(*χ*_*o*_, *τ*) > *θ*_*c*_. Thus, *χ*_*o*_ is still part of region 1 (Figure 5) where the pressure is approximately uniform at high wall stiffness and low fluid viscosity. This is because, in this case, the dynamic pressure gradients (pressure changes due to fluid motion) are not dominant in the momentum equation. Consequently, although CSA decreases, pressure remains approximately constant $\left(p_{\chi_{o}}\approx p_{1}\right)$ in phase **(a)**.

Dynamic pressure gradients do become relevant for low tube stiffness or high viscosity (to overcome fluid friction). In this case, pressure gradients develop within region 1 (Figure 5) and one can see that, although minor compared to phase **(a)**, $p_{\chi_{o}}$ increases slightly during phase **(b)** (Figure 6A). However, this scenario is not expected in clinical FLIP data as the esophagus stiffness is relatively high and fluid (saline) viscosity is low.

(c) In this phase, point *χ*_*o*_ transitions from being proximal to being distal to the contraction (point *c*). At the middle of phase **(c)**, *θ*(*χ*_*o*_, *τ*) = *θ*_*c*_ and CSA at *χ*_*o*_ is minimum. Around the minimum area the viscous resistance for the fluid to flow from region 1 to region 2 is maximum. The pressure gradient across the minimum area provides the force necessary to overcome the viscous resistance. Thus, $p_{\chi_{o}}$ decreases from $p_{\chi_{o}}\approx p_{1}$ to $p_{\chi_{o}}\approx p_{2}$ . Pressure drops faster than CSA changes in phase **(c)**, so this transition occurs at almost constant CSA.
(d) As the contraction wave exits location *χ*_*o*_, *θ*(*χ*_*o*_, *τ*) increases, which implies that CSA increases. However, *χ* = *χ*_*o*_ is proximal to the contraction and in region 2 (Figure 5). For reasons similar to those discussed in phase **(b)**, the pressure is almost uniform in region 2. Consequently, $p_{\chi_{o}}\approx p_{2}$ and phase **(d)** takes place at constant pressure. Note that in some clinical contraction cycles, such as the one plotted in Figure 2C, phase **(d)** is less horizontal, and pressure decreases as CSA increases. This is because, in contrast to the activation function defined in the simulations, the contraction strength in the actual esophagus is not constant, and may decrease throughout the contraction cycle. Hence, the muscle activation which pressurizes the tube decreases, which slightly reduces the pressure in the tube.
(e) The constant pressure during phase **(d)** is lower than the initial pressure. In phase **(e)**, the pressure at *χ* = *χ*_*o*_ increases back to the initial pressure. As the contraction wave reaches the closed distal end, and eventually exits the domain, it allows more back-flow, causing the displaced fluid to flow upstream. This enhanced back-flow increases the pressure at point *χ*_*o*_, as the tube returns to its initial state. Note that in clinical data, the contraction is a repetitive process, meaning that as the advancing contraction wave exits at the distal end of the tube, another peristaltic wave enters the domain at the proximal end (Carlson et al., 2020). Given this set up, it is hard to separate phases **(a)** and **(e)**, and the loop looks like one continuous process.

An additional complication is introduced when the FLIP bag is placed such that it crosses the LES. As the peristaltic contraction cycle starts, (phase **(a)**), the LES muscle begins to relax, as illustrated by the activation function in Figure 3B. Thus, the pressure in the tube is controlled by LES tone and the peristaltic contraction, each mechanism affecting the pressure at *χ* = *χ*_*o*_ differently. Peristaltic wave increases pressure distal of the contraction. Sphincter relaxation leads to pressure drop. Figure 6C presents the resulting P-CSA loop of such a simulation. As it turns out, although both mechanisms are present and cause different outcomes in pressure, the resulting loop has the same horizontal pattern. Most importantly, the pressure at point *χ* = *χ*_*o*_ during phase **(a)** increases. The reason for that was explained in a prior work (Elisha et al., 2021), where the P-CSA hysteresis loop at the LES was plotted. Their study revealed that pressure and CSA at the LES increase and decrease together. That is, as the LES opens, the pressure at the LES increases. While originally counter intuitive, this P-CSA relation was explained by Elisha et al. (2022b). The LES opening is a superposition of two mechanisms; neurogenic mediated relaxation of the LES muscle, and peristaltic contraction that causes pressurized distention. Thus, although the LES opens, which in an isolated case would reduce the pressure in region 1, the pressure increase from the peristaltic contraction dominates.

With this understanding in mind, the parallel between the horizontal loops in Figures 6A, C is clear. In phase **(a)**, as the peristaltic wave travels down the deformable tube, the pressure and therefore the CSA increases distal to the contraction. Although the LES opens, the pressure rise from the traveling contraction dominates such that the pressure increases in phase **(a)**. Note that this pressure increase is not as high as in the case where the FLIP does not pass through the LES (in Figure 6A).

By the time the contraction wave reaches point *χ*_*o*_, marking the beginning of phase **(b)**, the LES is fully open and remains open for the rest of the cycle. Hence, phases **(b)**, **(c)**, **(d)** are analogous to the ones described for Figure 4.

Lastly, note that the P-CSA loop plotted by Gregersen et al. (2011) and presented in Figure 2A has a slightly different shape than the remaining clinical cases presented in Figure 2. This loop does not have a well defined phase **(a)**. Since the FLIP length is the main parameter that differentiates the case in Figure 2A from the rest, we hypothesize that the FLIP length causes this difference. To test this hypothesis, we simulate flow inside a short FLIP placed in the esophagus by changing the contraction width (w) from .25 (as in the case in Figure 6A) to w = .75. The P-CSA loop from this simulation is displayed in Figure 6D. It is seen that decreasing the tube length conserves the general horizontal orientation of the loop but eliminates the original phase **(a)**. The resulting loop has a more drop-like shape, similar to the corresponding clinical loop by Gregersen et al. (2011). The reason for that is simple: the wave hardly travels outside point *χ*_*o*_ because the width of the contraction is almost equal to the width of the FLIP bag.

## 4 Conclusion

The goal of this study was to provide a mechanistic understanding of the P-CSA loops within the esophageal body. We examined the P-CSA hysteresis loop within the esophageal body obtained from clinical FLIP testing of various configurations. The considered cases were 1) 16-cm long FLIP bag placed inside the esophagus but did not pass through the LES, 2) 16-cm long FLIP bag which crossed the LES, and 3) an experimental setup reported by Gregersen et al. (2011), in which a 4-cm long FLIP bag was placed in the esophageal body, above the LES. The clinical P-CSA plots revealed a horizontal loop shape.

Similar to cardiovascular analyses, we divided the P-CSA loop into phases. However, we aimed to offer a new mechanics-based understanding of the different phases in the P-CSA loop within the esophageal body. To obtain this understanding, we simulated flow inside a FLIP device placed inside the esophagus lumen. This gave us a closer look into the esophageal mechanism at each time instant. This information allowed us to develop a clear view of the phases of peristaltic contraction inside the FLIP. We concluded that the horizontal loop pattern within the esophagus body and the phases are a product of different mechanical conditions (muscle contraction, boundary condition, and geometric configuration) rather than any inherently different function of the muscle itself.

## Data Availability

The raw data supporting the conclusion of this article will be made available by the authors upon request, without undue reservation.
